# Muscle biopsy practices in the evaluation of neuromuscular disease: A systematic literature review

**DOI:** 10.1111/nan.12888

**Published:** 2023-02-20

**Authors:** Laura Ross, Penny McKelvie, Katrina Reardon, Huon Wong, Ian Wicks, Jessica Day

**Affiliations:** ^1^ Department of Rheumatology St Vincent's Hospital Melbourne Fitzroy Victoria Australia; ^2^ Department of Medicine The University of Melbourne at St Vincent's Hospital Fitzroy Victoria Australia; ^3^ Department of Anatomical Pathology St Vincent's Hospital Melbourne Fitzroy Victoria Australia; ^4^ Department of Neurology St Vincent's Hospital Melbourne Fitzroy Victoria Australia; ^5^ Inflammation Division Walter and Eliza Hall Institute of Medical Research Parkville Victoria Australia; ^6^ Department of Rheumatology Royal Melbourne Hospital Parkville Victoria Australia; ^7^ Department of Medical Biology University of Melbourne Parkville Victoria Australia

**Keywords:** diagnosis, muscle biopsy, myopathy, neuromuscular disease

## Abstract

**Aims:**

Muscle biopsy techniques range from needle muscle biopsy (NMB) and conchotome biopsy to open surgical biopsy. It is unknown whether specific biopsy techniques offer superior diagnostic yield or differ in procedural complication rates. Therefore, we aimed to compare the diagnostic utility of NMB, conchotome and open muscle biopsies in the assessment of neuromuscular disorders.

**Methods:**

A systematic literature review of the EMBASE and Medline (Ovid) databases was performed to identify original, full‐length research articles that described the muscle biopsy technique used to diagnose neuromuscular disease in both adult and paediatric patient populations. Studies of any design, excluding case reports, were eligible for inclusion. Data pertaining to biopsy technique, biopsy yield and procedural complications were extracted.

**Results:**

Sixty‐four studies reporting the yield of a specific muscle biopsy technique and, or procedural complications were identified. Open surgical biopsies provided a larger tissue sample than any type of percutaneous muscle biopsy. Where anaesthetic details were reported, general anaesthesia was required in 60% of studies that reported open surgical biopsies. Percutaneous biopsies were most commonly performed under local anaesthesia and despite the smaller tissue yield, moderate‐ to large‐gauge needle and conchotome muscle biopsies had an equivalent diagnostic utility to that of open surgical muscle biopsy. All types of muscle biopsy procedures were well tolerated with few adverse events and no scarring complications were reported with percutaneous sampling.

**Conclusions:**

When a histological diagnosis of myopathy is required, moderate‐ to large‐gauge NMB and the conchotome technique appear to have an equivalent diagnostic yield to that of an open surgical biopsy.

## Graphical abstract

A variety of muscle biopsy techniques have been reported in the literature. In this systematic review, muscle biopsies performed with a moderate‐ to large‐gauge needle or conchotome forceps were found to have equivalent clinical utility to open surgical biopsies.

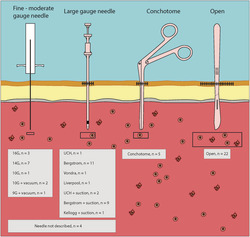

**Key points**

- Muscle biopsy remains an important diagnostic test for patients with muscle weakness of unknown aetiology.
- Various muscle biopsy techniques exist, including needle muscle biopsy, conchotome muscle biopsy and open surgical biopsy. The diagnostic equivalence of each biopsy technique has not previously been compared.
- The moderate‐ to large‐gauge needle and conchotome biopsies have an equivalent diagnostic yield to an open surgical biopsy, with the advantage of requiring only local anaesthesia, with or without light sedation.
- Muscle biopsies, irrespective of the technique, are safe and well tolerated with few adverse events reported.

## INTRODUCTION

A muscle biopsy has long been considered the cornerstone of the diagnosis of myopathy [1]. Despite advances in serological and genetic evaluation of myopathies, histopathological evaluation of skeletal muscle remains an important diagnostic test in patients with quantifiable weakness of uncertain aetiology [2]. Evaluation of muscle tissue through biopsy may permit a specific diagnosis, support or exclude a diagnosis made on clinical grounds and provide invaluable material for functional studies, molecular analyses or biobanking. Muscle biopsies are generally targeted to muscles that are suspected to be affected by disease; either because of clinical weakness, evidence of active myopathy on electromyography (EMG) studies or imaging changes detected by ultrasound (US) or magnetic resonance imaging (MRI) [2]. Deltoid, biceps and quadriceps muscles are commonly biopsied, as established norms for fibre type percentages and muscle fibre size of these muscle groups exist [2]. The volume of muscle tissue obtained in a given biopsy sample is important because a yield of at least 200–250 muscle fibres in a well‐oriented transverse section is generally required to confidently diagnose or exclude a myopathic process on histological grounds [3]. Sample volume is also an important consideration for nonmorphological diagnostic tests, such as mitochondrial studies.

**FIGURE 1:**
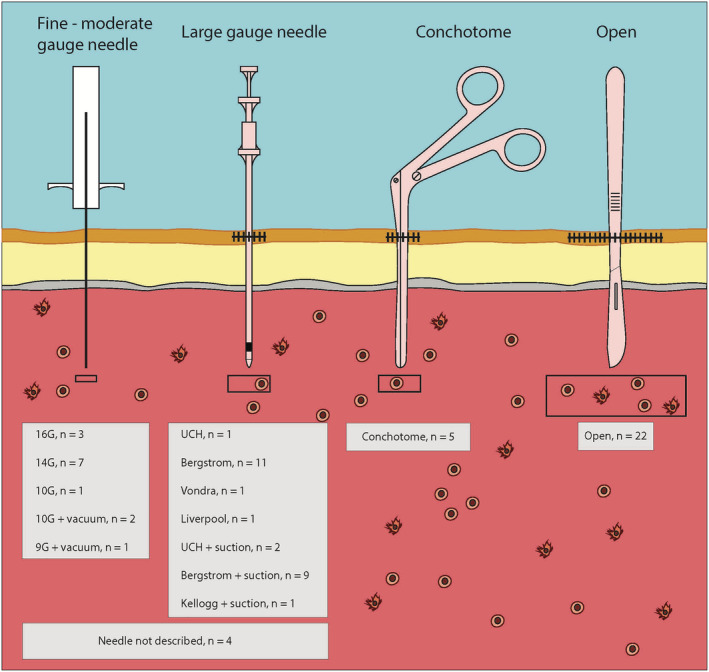
Types of muscle biopsy. *Note*: *n* = number of studies of each muscle biopsy technique included in this review.

Various muscle biopsy techniques exist (Figure 1). Based upon institutional experience, preference is given to either open surgical biopsy, needle muscle biopsy (NMB) or conchotome biopsy. Open muscle biopsies require a surgical team, with or without general anaesthesia, and an incision through the skin, subcutaneous tissue and muscle fascia, to obtain a sample that is characteristically 1 cm × 0.5 cm in size [2]. Needle muscle biopsies are performed with needles of various gauges, commonly under local anaesthesia or light sedation, and can be performed at the bedside. A specialised muscle biopsy needle, the Bergström needle was developed in the 1960s, when percutaneous NMB was reintroduced to routine clinical practice [4]. This needle, constructed of two parallel cylinders of up to 5 mm in diameter, can yield sufficient muscle tissue without the need for an open incision. While NMB has the advantage of not requiring general anaesthesia or a large incision, the tissue yield of an NMB is smaller [5]. Insufficient yield of tissue from an NMB has been considered a limitation of this procedure, leading to the introduction of suction NMB to increase the volume of tissue obtained with each procedure [3]. The Well–Blakesley conchotome forceps (alligator forceps that open with a scissor grip [3]) are an alternative to the Bergström needle for percutaneous muscle biopsy, again allowing for bedside muscle biopsy under local anaesthesia. A conchotome biopsy is thought to yield a similar volume of tissue as an NMB, but the conchotome forceps may allow for more precise placement of the forceps compared with percutaneous needle puncture, which may be advantageous in diagnosing focal rather than diffuse myopathic changes [3].

The diagnostic equivalence of various muscle biopsy techniques has not been systematically compared. Given the potential benefits of an NMB or conchotome biopsy, it is of clinical importance to establish whether an NMB has equivalent diagnostic utility to an open surgical biopsy. Therefore, we performed a systematic literature review of NMB, conchotome and open muscle biopsies, both in relation to the volume of muscle tissue obtained and the diagnostic yield of each procedure.

## METHODS

This study was performed in accordance with the Preferred Reporting Items for Systematic Reviews and Meta‐Analysis (PRISMA) checklist [6]. A systematic literature search of the EMBASE and Medline (Ovid) databases from January 1970 to July 2021 was performed to identify original research articles that described the muscle biopsy technique used to diagnose neuromuscular disease in either adult or paediatric patients. Keywords used in the search were (muscle, muscles or muscular), (biopsy, biopsies, microbiops*), (percutaneous or needle or needles), conchotome (diagnosis or diagnostic or pathology or sensitivity or specificity or classification or safety or complication).

After de‐duplication, all retrieved abstracts were reviewed by a single author (JD) to identify relevant studies for full‐text review. If there was any uncertainty about study eligibility, it was included for full‐text review. Two authors (LR and JD) independently reviewed the full text of all eligible abstracts. Each author independently assessed the eligibility of all full‐text articles and uncertainty was resolved by consensus. Studies were eligible for inclusion if they were original research articles that provided information about muscle biopsy technique and described the muscle biopsy sample size, diagnostic yield or complications of the procedure. Human studies of any neuromuscular condition published in English were eligible for inclusion. Studies were excluded if combined nerve and muscle biopsies were performed. Studies including both adult and paediatric populations were eligible. Case reports and scientific meeting abstracts were excluded from the review. The same two authors independently extracted information regarding the study design and patient population, muscle biopsy technique and biopsy yield and procedure complications according to a prespecified template (see Data S1). An open surgical muscle biopsy was defined as a biopsy requiring an incision through the skin and subcutaneous tissue and excision of muscle tissue using a scalpel. A fine‐needle biopsy (FNB) was defined as a percutaneous diagnostic procedure performed using a 14‐ to 16‐gauge needle. A moderate‐gauge NMB was defined by the use of a 9‐ to 10‐gauge needle, and a large‐gauge NMB was defined by the use of <9‐gauge needle to perform a percutaneous procedure. Descriptive statistics were used to present the study results. Owing to the heterogeneity of study methodology and outcome, it was not possible to perform a meta‐analysis of the data extracted. Abstracts were screened using the citation management software Covidence (www.covidence.org), and full‐text articles were managed using the bibliographic manager EndNote X9.3.3 (Thomas Reuters). Ethical approval was not required for this study.

## RESULTS

**FIGURE 2:**
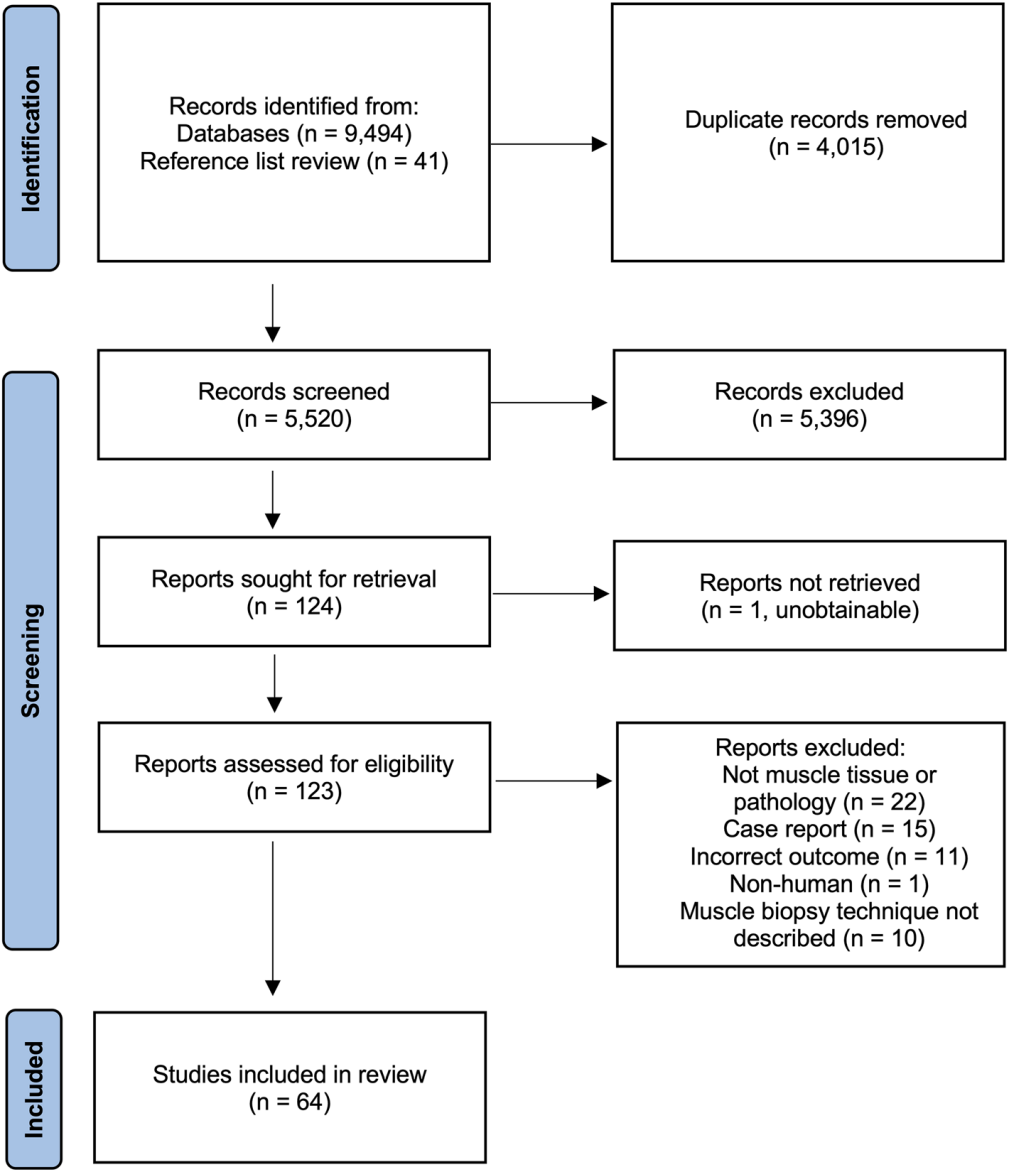
PRISMA flowchart of study selection process.

The search identified 9536 citations which after de‐duplication left 5479 references for review. The full text of 124 studies was reviewed. A further 41 references were identified through manual searching of study reference lists. A total of 64 studies met the prespecified criteria for inclusion in the final review (Figure 2). The study characteristics of all studies included in the final review are detailed in Table 1 and Figure 1.

**TABLE 1:** Study characteristics.

Author Year Country	Study type	Diagnosis/suspected diagnosis	Inclusion criteria	Population	Mean age (years) (range)	Number of patients	Number of biopsies	Biopsy type	Biopsy setting	Incision size (cm)	Anaesthesia used
Open surgical biopsy (OSB)											
Aburahma [7] 2019 USA	Retrospective case series	Myopathy	Repeat muscle biopsy	A	16 (18–80)	78	143	OSB	Operating theatre	NR	General anaesthesia
Buchthal [8] 1982 Denmark	Retrospective case series	Neuromuscular disorders	Consecutive muscle biopsies	A/P	NR	188	348	OSB	NR	NR	NR
Constantinides [9] 2018 Greece	Retrospective case series	Documented EMG and biopsy data	Suspected myopathy	NR	NR	123	123	OSB	NR	NR	Local anaesthesia
Gibertoni [10] 1987 Italy	Case series	Neuromuscular disorders	Consecutive patients	NR	NR	53	NR	OSB	NR	NR	NR
Gibreel [11] 2014 USA	Retrospective case series	Neuromuscular disorders	Consecutive surgical muscle biopsies	P	7 (9 days to 18 years)	169	169	OSB	Operating theatre	NR	General anaesthesia
Goutman [12] 2013 USA	Retrospective case series	Myopathy	Consecutive patients undergoing repeat muscle biopsy	A/P	Median: 41.6 (0.48–79.4)	66	149	OSB	NR	NR	NR
Jamshidi [13] 2008 USA	Retrospective case series	Neuromuscular disorders	Consecutive biopsies	P	5.3 (8 days to 21 years)	127	127	OSB	NR	NR	NR
Kokotis [14] 2016 Greece	Retrospective case series	Elevated creatine kinase for investigation	Asymptomatic elevated serum creatine kinase	A	18–76	19	10	OSB	NR	NR	Local anaesthesia
Laguno [15] 2002 Spain	Retrospective case–control study	Muscle disorders of elderly	Consecutive muscle biopsies from individuals >65 years Control group: biopsies in patients <65 years	A	Cases: 72.1 ± 5 years Controls: 40 ± 16 years	239	478	OSB	Outpatient clinic	NR	Local anaesthesia
Lai [16] 2010 USA	Retrospective case series	Myopathy	Consecutive muscle biopsies; excluding autopsy studies or combined muscle and nerve biopsy	A/P	47 ± 22 (2 weeks to 84 years)	258	258	OSB	NR	NR	NR
Reynolds [17] 1999 USA	Retrospective case series	Neuromuscular disorders	Consecutive muscle biopsies	P	NR	153	153	OSB	Operative theatre	2–3 cm	General anaesthesia
Shaibani [18] 2015 USA	Retrospective case series	Myopathy	Consecutive muscle biopsy	A	55.32 ± 15.55	698	720	OSB	NR	NR	NR
Shapiro [19] 2016 USA	Retrospective case series	Neuromuscular disorders	Consecutive muscle biopsy	P	<1 month to 19 years	877	877	OSB	Operative theatre	1.2–1.8 cm	General anaesthesia
Sujka [20] 2018 USA	Retrospective case series	Neuromuscular disorders	Consecutive muscle biopsy	P	Median: 5 (2–10)	90	90	OSB	NR	NR	NR
Tenny [21] 2018 USA	Retrospective case series	Myopathy	Consecutive muscle biopsy	A	57.4 ± 16.9 Range: 21–86	106	106	OSB	Operative theatre	NR	NR
Thavorntanaburt [22] 2018 Thailand	Retrospective case series	Neuromuscular disorders	Consecutive muscle biopsy	P	7.1 ± 4.2	92	94	OSB	Operating theatre	2 cm	General anaesthesia
Van de Vlekkert [23] 2015 The Netherlands	Prospective cohort study	Idiopathic inflammatory myositis	Suspected subacute inflammatory myositis	A	50 ± 14	48	47	OSB	NR; MRI triage of biopsy site	NR	Local anaesthesia
Yang [24] 2019 USA	Retrospective case series	Neuromuscular disorders	Consecutive muscle biopsy	P	7.7	220	220	OSB	Operating theatre	NR	General anaesthesia
Fine‐needle biopsies											
Agten [25] 2018 Belgium	Prospective pilot study	Chronic low back pain	Nonspecific chronic low back pain	A	45.60 ± 8.81	15	30	16G NMB	Ultrasound landmarking of biopsy site	Needle puncture	Local anaesthesia
Tobina [26] 2009 Japan	Prospective case series	Feasibility study	Research volunteers	A	Young males: 23.8 ± 2.3 Older males: 52.9 ± 7.7 Older females: 61.1 ± 8.4	40	40	16G NMB	NR	Needle puncture	Local anaesthesia
Campellone [27] 1997 USA	Retrospective case series	Idiopathic inflammatory myositis	All patients with suspected IIM	A	NR	55	66	14G NMB	Hospital ward, EMG laboratory	Needle puncture	Local anaesthesia
Cote [28] 1992 USA	Retrospective case series	Neuromuscular disorders	Consecutive muscle biopsies	A/P	NR	105	NR	14G NMB	NR	Needle puncture	Local anaesthesia
Lindequist [29] 1990 Denmark	Retrospective case series	Neuromuscular disorders	Consecutive muscle biopsies	A/P	Males: 37.7 Females: 39.7	24	28	14G NMB	Ultrasound targeted biopsy	‘Small’	Local anaesthesia
Magistris [30] 1998 Switzerland	Retrospective case series	Neuromuscular disorders	Consecutive muscle biopsies	A/P	NR	220 (211 adults, 9 children)	220	14G NMB	NR	0.1–0.2 cm	Local anaesthesia; Sedation for 3 children
Paoli [31] 2010 Italy	Prospective pilot study	Feasibility study	Healthy volunteers	A	NR	18	18	14G NMB	NR	Needle puncture	Local anaesthesia
Moderate‐gauge needle biopsies											
Barthelemy [32] 2020 USA	Retrospective case series	Duchenne muscular dystrophy or Becker muscular dystrophy	Healthy individuals and individuals with Duchenne muscular dystrophy or Becker muscular dystrophy	A/P	12.9 (2–66)	94	471	10G NMB with vacuum	Research setting, clinic, hospital ward, OT; Ultrasound guided	0.3 cm	Local anaesthesia Sedation with IV fentanyl and propofol (children only)
Bylund [33] 1981 Sweden	Retrospective case series	Compartment syndrome	NR	NR	NR	12	36–48	10G NMB	NR	0.3–0.4 cm	Local anaesthesia
Gallo [34] 2018 Canada	Retrospective case series	Neuromuscular disorders	Consecutive percutaneous muscle biopsies	A	55 (17–88)	92	102	10G NMB with vacuum	NR	1 cm	Local anaesthesia
Lassche [35] 2018 The Netherlands	Case series	FSHD	Genetically confirmed FSHD and enrolled in separate clinical study	NR	NR	13	12	9G NMB with vacuum	MRI guided; MRI table	0.4 cm	Local anaesthesia
Large‐gauge needle biopsies											
Cotter [36] 2013 USA	Prospective case series	Feasibility study	Participants enrolled in exercise study	A	21.4 ± 2.9	45	84	6G UCH NMB with suction	Outpatient clinic	0.5–0.6 cm	Local anaesthesia
Derry [37] 2009 United Kingdom	Retrospective case series	Myopathy and neuromuscular disorders	Consecutive muscle biopsies	A/P	Median age: 51 (1–86)	870	900	6 mm Bergström NMB	Bedside	1 cm	Local anaesthesia
Di Liberti [38] 1983 USA	Retrospective case series	Neuromuscular disorders or Reye syndrome	Consecutive muscle biopsies	P	1 week to 15 years	77	86	3 to 5 mm Bergström NMB	Outpatient clinic, ward	0.5–0.8 cm	Local anaesthesia Sedation if age <8 years
Edwards [39] 1973 United Kingdom	Retrospective case series	Myopathy	Patients with muscular symptoms	A/P	42.4 (10–68)	31	32	4.5 mm Bergström NMB	Outpatient clinic, ward	0.4–0.5 cm	Local anaesthesia
Evans [40] 1982 USA	Prospective pilot study	Volunteers from clinical study	NR	A	NR	30	60	4–5 mm Bergström NMB with suction	NR	NR	NR
Hennessey [41] 1997 USA	Case series	NR	Volunteers from human growth hormone in frail elderly study	A	NR	46	83	4–6 mm Bergström NMB with suction	NR	0.6–0.9 cm	Local anaesthesia
Highstead [42] 2005 USA	Retrospective case series	Healthy individuals	Healthy volunteers	A	18–76 years	161	1301	5 mm Bergström NMB with suction	Research laboratory	2 cm	Local anaesthesia
Iachettini [43] 2015 Italy	Prospective case–control	Myotonic dystrophy type I	Known patients and healthy controls	A	Patients: 29.8 (21–42)	5 patients 5 controls	10	5 mm UCH NMB with suction	NR	1 cm	Local anaesthesia
Kirby [44] 1982 Canada	Prospective cohort study	Healthy athletes & clinically suspected muscle disease	Consecutive biopsies	A/P	6–79 years	60	444	4–5 mm Bergström NMB; various manoeuvres to fill needle window performed	NR	0.5–0.7 cm	Local anaesthesia
Leong [45] 1993 Singapore	Case series	Myopathy	Consecutive muscle biopsies	A	44 (range 20–69)	24	24	Bergström NMB	NR	NR	Local anaesthesia
Maunder‐Sewry [46] 1981 United Kingdom	Prospective case–control study	Duchenne muscular dystrophy	Genetically obligate carriers, possible carriers, healthy volunteers	A/P	2–61	65	75	Bergström NMB	NR	NR	Local anaesthesia
Melendez [47] 2007 USA	Case series	No neuromuscular disease	Volunteers from other clinical studies	A	50 ± 4.5	55	55	6 mm Bergström NMB with suction	NR	3 cm	Local anaesthesia
Mubarak [48] 1992 USA	Retrospective case series	Neuromuscular disorders	Consecutive percutaneous NMB	A/P	1 week to 75 years 92% aged <18 years	379	379	5 mm Bergström NMB with suction	≥12 years: outpatient clinic <12 years: operating theatre	‘Small stab wound’	≥12 years: local anaesthesia <12 years: general anaesthesia
Neves [49] 2012 Brazil	Retrospective case series	No neuromuscular disease	Clinical study participants Healthy volunteers (*n* = 168) Individuals with chronic illness (*n* = 106)	A	Healthy subjects: 24 ± 8 years Subjects with chronic illness: 55 ± 8 years	274	496	5 mm Bergström NMB with suction	NR	NR	Local anaesthesia
Pamphlett [50] 1985 Australia	Retrospective case series	Neuromuscular disorders	Consecutive NMB	A/P	9–75	70	75	5 mm Kellogg NMB with suction	NR	0.5 cm	Local anaesthesia
Raithatha [51] 2020 United Kingdom	Retrospective case series	Myopathy	Consecutive patients unable to have a surgical biopsy	NR	NR	10	11	5 mm Bergström NMB with suction	Ultrasound guided, MRI triage of the biopsy site	NR	Local anaesthesia
Schwarz [52] 1980 United Kingdom	Retrospective case series	Idiopathic inflammatory myositis	Consecutive muscle biopsies	A/P	7–81 years	30	30	Bergström NMB	NR	0.5 cm	NR
Sengers [53] 1980 The Netherlands	Retrospective case series	Muscle disease, cardiomyopathy, other	Consecutive muscle biopsies	P	2 months to 17.5 years	95	98	Vondra NMB	Ward, outpatient clinic	0.3 cm	Local anaesthesia
Tarnopolsky [54] 2011 Canada	Retrospective case series	Myopathy	Consecutive muscle biopsy	A/P	NR	NR	13,914	5 mm Bergström NMB with suction	NR	0.4–0.5 cm	Local anaesthesia
Wang [55] 2019 USA	Prospective case–control study	FSHD	Genetically confirmed FSHD (*n* = 33) and healthy controls (*n* = 9)	A	54 (20–75)	42	42	UCH or Bergström NMB	NR	NR	NR
Needle size not described											
Billakota [56] 2016 USA	Prospective pilot study	Myopathy	Consecutive patients referred for muscle biopsy	A	55.7 ± 15.7	40	NR	NMB; size not described	Ultrasound landmarking of biopsy site	NR	NR
Curless [57] 1975 USA	Retrospective case series	Neuromuscular disorders	Children <5 years	P	9 weeks to 3 years	14	15	NMB; size not described	NR	0.5 mm	Sedation (chloral hydrate)
Hafner [58] 2019 United Kingdom	Retrospective case series	Neuromuscular disorders	Definite or probable myopathy or neurogenic disorder or no neuromuscular disorder diagnosed	P	6.8	171	171	NMB; size not described	NR	NR	General anaesthesia
Conchotome muscle biopsies (CMBs)											
Dorph [59] 2001 Sweden	Retrospective case series	Idiopathic inflammatory myositis	Consecutive muscle biopsies	A/P	Median: 56 (16–85)	122	149	CMB	Outpatient clinic, ICU	0.5–1 cm	Local anaesthesia
Henriksson [60] 1979 Sweden	Retrospective case series	Neuromuscular disorders	Consecutive muscle biopsies	A/P	<1–89 years	959	959	CMB	NR	0.3–0.5 cm	Local anaesthesia
Patel [61] 2011 United Kingdom	Prospective cohort study (feasibility study)	Sarcopenia	Male participants in Hertfordshire Cohort Study **ref**	A	72 (68–77)	102	102	CMB	Research laboratory (overnight stay)	0.5–1 cm	Local anaesthesia
Poulsen [62] 2005 Denmark	Retrospective case series	Neuromuscular disorders	Consecutive muscle biopsies	A/P	10–81	157	160	CMB	NR	1–2 cm	Local anaesthesia and IM opiate analgesia
Comparative studies											
Dengler [63] 2014 Germany	Prospective case–control study	Amyotrophic lateral sclerosis	Participants in adjunct clinical study	A	63 ± 8	33	66	5 mm Bergström NMB vs OSB	Operating theatre	0.5 cm NMB 3–4 cm OSB	Local anaesthesia
Dietrichson [64] 1987 United Kingdom	Retrospective case series	Neuromuscular disorders	Consecutive muscle biopsies	A/P	32.4 (8 days to 70 years)	292	436	CMB (*n* = 222) vs Liverpool NMB (*n* = 330)	Ward, outpatient clinic, ICU, research laboratory	CMB: 0.5 cm NMB: NR	Local anaesthesia
Fukuyama [65] 1981 Japan	Retrospective case series	Neuromuscular disorders	Consecutive muscle biopsies	A/P	2 months to 35 years	75	NMB: 3–4 biopsies/patient Open: 1 biopsy/patient	OSB (*n* = 33) vs 14G NMB (*n* = 75)	NR	NMB: 0.1 cm OSB: NR	NMB: local anaesthesia Open: general anaesthesia
Greig [66] 1985 USA	Retrospective case–control	NR	Consecutive muscle biopsies	NR	NR	37	84	5 mm Bergström NMB with suction vs 5 mm Bergström NMB without suction	NR	0.6–1.3 cm	Local anaesthesia
Hayot [67] 2005 Canada	Prospective cohort study	COPD	COPD confirmed by RFT and healthy controls	A	COPD: 69 ± 5 Controls: 27–29	17 COPD 4 controls	42	16G NMB vs 6 mm Bergström NMB	Exercise physiology laboratory	16G NMB: needle puncture Bergström NMB: 1 cm	Local anaesthesia
Heckmatt [68] 1984 United Kingdom	Retrospective case series	Neuromuscular disorders	Consecutive NMB over a 5‐year period	A/P	Children: 1 day to 17 years	700	700	5 mm Bergström NMB vs OSB (*n* = 24)	Ward, outpatient clinic	NR	Local anaesthesia Sedation with chloral hydrate for children aged 12 months‐8 yrs. No sedation if age <12 months or >8 years
O′Rourke [69] 1994 USA	Retrospective case–control study	Neuromuscular disorders	Consecutive NMB and open muscle biopsy	A	NMB: 45.9 Open: 52.5 Range: 19–84	121	121	NMB (*n* = 30); size not described vs OSB (*n* = 91)	NR	NMB:0.5 cm OSB: NR	NMB: Local anaesthesia Some patients received IV midazolam sedation Open: NR
O′Sullivan [70] 2006 Ireland	Prospective case series (NMB) vs historical controls (OSB)	Neuromuscular disorders	Consecutive referrals for muscle biopsy	A	56 (35–70)	80	80	14G NMB (*n* = 40) vs OSB (*n* = 40)	Ultrasound guided, Radiology Department	NMB: 0.3–0.4 cm OSB: NR	Local anaesthesia

*Note*: Grey shaded boxes = data not reported in the study.

Abbreviations: A, adult study population; CMB, conchotome muscle biopsy; EMG, electromyography; FSHD, facioscapulohumeral muscular dystrophy; ICU, intensive care unit; IM, intramuscular; IV, intravenous; MRI, magnetic resonance imaging; NMB, needle muscle biopsy; NR, not reported; OSB, open surgical biopsy; P, paediatric study population; UCH, university college hospital; USA, United States of America.

Studies considered the role of muscle biopsy in the evaluation of suspected neuromuscular disorders (*n* = 26), myopathy not further specified (*n* = 13), muscular dystrophies (*n* = 5), idiopathic inflammatory myositis (*n* = 4), healthy volunteers (*n* = 8) and other diagnoses (*n* = 8). Most studies (*n* = 43 [67%]) were retrospective analyses. Data from adult patients were presented in 48 (75%) studies. Paediatric data were presented in 32 (43%) studies. Twenty‐two (34%) studies reported open surgical biopsy results, 26 (41%) reported large‐gauge NMB results, 10 (16%) studies reported results of FNB, four (6%) studies reported moderate‐gauge NMB results and five (8%) studies reported conchotome biopsy findings. Eight (13%) studies compared two different biopsy techniques. Ten open surgical biopsies studies reported the type of anaesthesia used, with general anaesthesia used in 6/10 (60%) studies. Of these six studies, five were in a paediatric population and thus only one adult study reported the use of general anaesthesia during open biopsy. Needle biopsies and conchotome biopsies were generally performed under local anaesthesia (*n* = 40 [95%]) and commonly in outpatient or bedside settings.

### Muscle selection

**FIGURE 3:**
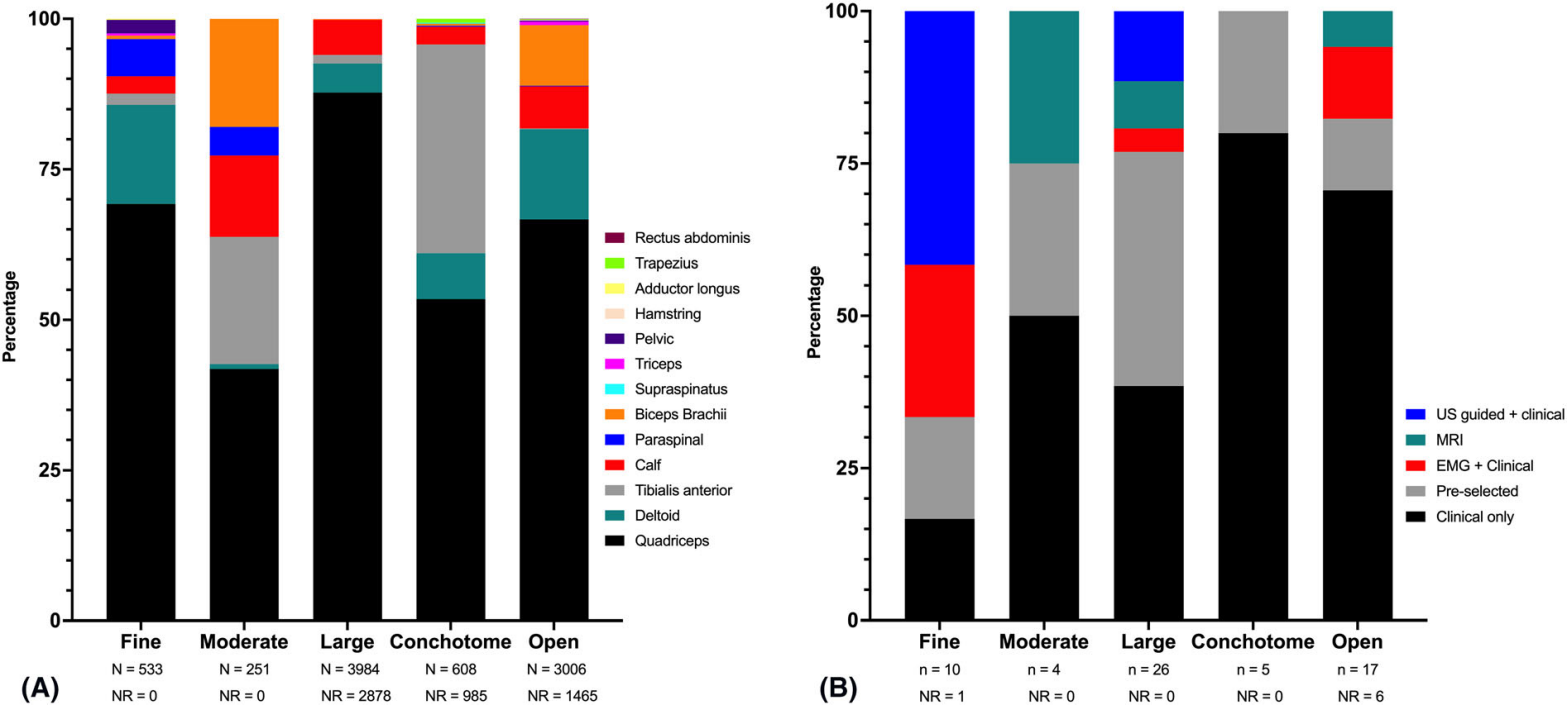
Site of muscle biopsy. (A) The range of muscles sampled using various muscle biopsy techniques. (B) Muscle selection strategies employed for various muscle biopsy techniques. ‘Fine’, ‘moderate’ and ‘large’ refer to needle biopsy size. Cohorts in which the needle size was not described have been excluded. *N*: number of biopsies; *n*: number of studies; NR: not reported.

Quadriceps muscles were the most commonly biopsied site, representing more than 50% of muscle biopsy sites for all types of muscle biopsy except moderate‐gauge NMB. The range of muscles sampled is illustrated in Figure 3A. Across all studies, 11 different muscle groups were sampled surgically, nine using FNB, eight using conchotome and seven using moderate‐ to large‐gauge needles. The site of muscle biopsy was selected based on imaging in only a minority of studies, with only three (4.69%) studies using MRI results to guide the selection of muscle biopsy sites and a further two (3.13%) studies using US to landmark the biopsy site. A further six (9.39%) studies used US to guide NMB for safety rather than muscle selection. An FNB was the most likely biopsy type to be image guided, typically using US (Figure 3B). The site of conchotome and open muscle biopsies performed as part of clinical care, outside of clinical trials, were selected based on clinical examination and EMG findings. There was little data evaluating the role of imaging to guide or inform muscle biopsies, based on radiological evidence of active myopathy. An uncontrolled series reported a diagnostic yield of 93% from US‐targeted biopsies [29]. The results from the single study to compare US‐targeted biopsies to those guided by clinical and EMG findings alone suggested US targeting of muscle sites was not superior to the selection of muscles on clinical and/or EMG findings alone [56]. Inclusion of MRI as part of the diagnostic evaluation for idiopathic inflammatory myopathies showed that sampling a muscle of intense T2‐weighted signal reduced the false negative biopsy rate to 0.19 compared with the overall cohort false negative biopsy rate of 0.23 [23]. One small study suggested a 100% diagnostic yield of biopsies from sites selected by positive MRI and additional targeting of biopsy site using US at the time of the procedure [51].

### Sample yield

Thirty‐eight studies quantified the yield of muscle tissue from the biopsy technique under investigation; however, results were variably reported (Table 2). Thirty‐nine studies reported the rate of inadequate tissue sampling to permit histological analysis. Not unexpectedly, the largest tissue samples were obtained from open surgical biopsies. There was a graded relationship between the size of the needle used for NMB and the frequency of inadequate tissue sampling. However, only FNB had an inadequate tissue sample frequency of more than 4%.

**TABLE 2:** Muscle biopsy sample yield.

Biopsy type	Sample yield			Rate of inadequate sampling for histological analysis	
	Volume	Fibres per section	Weight (mg)	Range	Percentage
Open [8, 9, 11, 14, 17, 18, 22, 24, 63, 65, 69, 70]	1–3 × 1 cm^3^ pieces *n* = 6 studies	NR	NR	0% to 5% *n* = 10 studies	4/1294 (0.3%)
Fine‐gauge needle [25, 26, 27, 28, 29, 30, 31, 65, 67, 70]	2 × 0.2 cm^3^ pieces *n* = 1 study	min: 144 (38–286)^a^ max: 500 *n* = 5 studies	min: 4.2 ± 2.7 mg^d^ max: 55 ± 17 mg^d^ *n* = 5 studies	0% to 15% *n* = 7 studies	68/598 (11.4%)
Moderate‐gauge needle; no vacuum [33]	NR	125 (80–40)^a^ *n* = 1 study	12–28 mg^b^ *n* = 1 study	NR	NR
Moderate‐gauge needle with vacuum [32, 34, 35]	NR	NR	min: 190 mg (80–500 mg)^c^ max: 377–550 mg^b^ *n* = 2 studies	0% to 8% *n* = 3 studies	8/242 (3.3%)
Large‐gauge needle; no vacuum [37, 38, 39, 40, 44, 45, 46, 52, 53, 55, 66, 67, 68]	NR	min: 400–1200^b^ max (infants): 5000–10,000^b^ max (adults): 1060–1350^b^ *n* = 4 studies	min: 37 ± 3 mg^e^ max: 217 ± 89 mg^d^ *n* = 8 studies	0% to 8.3% *n* = 9 studies	39/2312 (1.7%)
Large‐gauge needle with vacuum [36, 41, 42, 43, 47, 48, 50, 51, 54, 64]	2 × 0.5–1 cm^3^ pieces *n* = 1 study	425 (288–623)^c^ *n* = 1 study	min: 61.5 ± 15.7 mg^d^ max: 233 ± 41.6 mg^d^ *n* = 8 studies	0% to 8% *n* = 8 studies	29/14,027 (0.2%)
Conchotome [59, 60, 61, 62, 64]	NR	500 (100–2000)^c^ *n* = 1 study	min: 23–123 mg^b^ max: 500–1000 mg^b^ *n* = 5 studies	0% to 3% *n* = 4 studies	26/1500 (1.8%)

*Note*: Grey shaded boxes = data not reported for specific biopsy type.

Abbreviations: max, maximum; mg, milligrams; min, minimum; NR, not reported.

^a^ Median (IQR).

^b^ Range.

^c^ Mean (range).

^d^ Mean ± SD.

^e^ Mean ± SE.

### Diagnostic yield

Thirty‐four (53%) studies provided data on the diagnostic yield of the muscle biopsies (Table 3). Muscle biopsy findings contributed to a clinical diagnosis in 31% to 100% of procedures. The tests performed on muscle tissue were not standardised across studies, which may have contributed to the variable diagnostic yield observed. Only 11 studies reported isolating genetic material from muscle tissue for analysis; the majority of these were published after 2007 and performed targeted gene profiling [11, 12, 18, 24, 26, 30, 36, 43, 47, 54, 55]. There was no observed difference in the diagnostic yield of muscle biopsy between specific neuromuscular disorders.

**TABLE 3:** Diagnostic yield of muscle biopsy procedures.

Biopsy type	Frequency of diagnostic tests performed on muscle	Biopsy contributed to the diagnosis^a^	Specific pathological findings observed	Normal histological findings	Nonspecific or nondiagnostic findings observed
Open [8, 9, 10, 11, 13, 14, 15, 16, 17, 18, 19, 20, 21, 22, 23, 24, 69, 70]	MS and HC (100%) IHC (70%) EM (60%) Western blot (10%) Metabolic studies (30%)	34% to 91% *n* = 9 studies *N* = 1317 total biopsies	23% to 80% *n* = 10 studies *N* = 2573 total biopsies	9% to 41% *n* = 11 studies *N* = 1917 total biopsies	9% to 53% *n* = 13 studies *N* = 3038 total biopsies
Fine‐gauge needle [25, 26, 27, 28, 29, 30, 31, 65, 67, 70]	MS and HC (100%) IHC (0%) EM (33%) Western blot (0%) Metabolic studies (33%)	43% to 80% *n* = 4 studies *N* = 327 total biopsies	67% *n* = 1 study *N* = 55 biopsies	13% to 30% *n* = 2 studies *N* = 260 total biopsies	13% to 48% *n* = 4 studies *N* = 417 total biopsies
Moderate‐gauge needle with vacuum [34]	NR	38% *n* = 1 study *N* = 102 biopsies	NR	17% *n* = 1 study *N* = 102 biopsies	36% *n* = 1 study *N* = 102 biopsies
Large‐gauge needle; no suction [37, 39, 45, 50]	MS and HC (100%) IHC (0%) EM (100%) Western blot (0%) Metabolic studies (100%)	31% to 100% *n* = 2 studies *N* = 56 total biopsies	49% to 58% *n* = 2 studies *N* = 940 total biopsies	18% to 28% *n* = 3 studies *N* = 972 total biopsies	23% to 41% *n* = 2 studies *N* = 932 total biopsies
Large‐gauge with suction [51, 54]	MS and HC (100%) IHC (100%) EM (0%) Western blot (0%) Metabolic studies (0%)	54% to 91% *n* = 2 studies *N* = 164 total biopsies	35% *n* = 1 study *N* = 153 biopsies	8% *n* = 1 study *N* = 153 biopsies	9% to 37% *n* = 2 studies *N* = 164 total biopsies
Conchotome [59, 60, 62]	MS and HC (100%) IHC (100%) EM (50%)	NR	26% to 56% *n* = 3 studies *N* = 1268 total biopsies	21% to 34% *n* = 3 studies *N* = 1268 total biopsies	13% to 40% *n* = 3 studies *N* = 1268 total biopsies

*Note*: Grey shaded boxes = data not reported for specific biopsy type. Only studies that reported the diagnostic yield of initial diagnostic muscle biopsies are presented. Studies evaluating the yield of repeat muscle biopsies are excluded from this table.

Abbreviations: EM, electron microscopy; HC, histochemistry; IHC, immunohistochemistry; MS, morphological stains; *n*, number of studies; *N*, number of biopsies; NR, not reported.

^a^ All analyses performed on biopsied tissue, not limited to histological findings.

A comparison between the diagnostic yield of various muscle biopsy techniques was performed in eight studies. One study compared conchotome biopsy to a large‐gauge NMB, with both procedures performed in all study participants under local anaesthetic. Study results indicated equivalent diagnostic utility for both procedures but less pain resulting from a conchotome biopsy [64]. Two studies made a direct comparison between the diagnostic yield of NMB, one study comparing FNB and the second comparing large‐gauge NMB to open surgical biopsies performed in the same patient. These studies both demonstrated equivalent diagnostic information can be gained from both procedures [65, 68]. Retrospective chart review of the diagnostic yield of FNB compared with open biopsy at two different centres suggested a reduced sensitivity (80% to 83%) of FNB for the detection of neuromuscular disorders compared with open biopsy (95% to 98%). However, in these studies, both procedures were not performed in each patient, and the diagnostic yield of each respective procedure was calculated from tissue samples taken from different patients [69, 70].

The clinical utility of repeat muscle biopsy was investigated in single‐centre retrospective studies [7, 12, 59]. Repeat muscle biopsy secured a diagnosis in 24% of patients and supported treatment decisions in a further 45% of patients in one series [12]. A second case series found that 47% of repeat muscle biopsies demonstrated different findings compared with initial biopsy results [7]. Repeat biopsy findings were more likely to be clinically relevant if the procedure was performed in a patient with a definite abnormal and inflammatory initial biopsy, ongoing proximal muscle weakness without myalgia and if the follow‐up biopsy showed evidence of polymyositis or inclusion body myositis [7].

### Complications

Muscle biopsy, irrespective of the biopsy technique, was generally well tolerated with a complication rate of less than 3% for all complications except haematoma or ecchymosis (Table 4). Haematoma or ecchymoses were reported in up to one‐third of patients undergoing large‐gauge NMB [44]. However, these results are from a study that actively screened for postprocedure bleeding complications with US [44]. Twenty‐one (33%) studies that included 2713 biopsies reported zero complications from any type of muscle biopsy procedure. There were no complications reported in any studies that performed a moderate‐gauge NMB without vacuum. Wound infections were most commonly reported in open surgical muscle biopsy studies, affecting between 1% and 5% of biopsy sites [70] as compared with up to 0.5% of biopsy sites with large‐gauge NMB [42, 49, 54]. Where reported, surgical incisions were ≥2 cm in length for 75% of open biopsy studies [17, 19, 22, 63] and ≤1 cm for 89% of percutaneous biopsy studies [25, 26, 27, 29, 30, 31, 32, 33, 34, 35, 36, 37, 38, 39, 41, 42, 43, 44, 47, 48, 50, 52, 53, 54, 56, 57, 59, 60, 61, 62, 63, 64, 65, 66, 67, 69, 70]. Persistent adverse events following muscle biopsy were uncommonly reported, with a 3.1% rate of keloid scarring reported in one open surgical biopsy study [15], and persistent sensory disturbance at biopsy site reported in 0.03% to 1.8% NMB studies [27, 42, 54, 59]. Ongoing weakness was reported in 2.3% of patients in one study following conchotome biopsy [60].

**TABLE 4:** Complications of muscle biopsy.

	Open	Needle not described	Fine gauge	Moderate gauge with vacuum	Large gauge; no vacuum	Large gauge with vacuum	Conchotome
Haematoma or ecchymosis	2.3% *n* = 1 [15] *N* = 479	NR	1% to 2% *n* = 2 [28, 30] *N* = 318	23% *n* = 1 [35] *N* = 13	36% *n* = 1 [44] *N* = 11	0.01% to 1.4% *n* = 4 [42, 48, 49, 54] *N* = 16,090	0.6% to 1.2% *n* = 3 [59, 60, 62] *N* = 395
Bleeding	NR	NR	NR	0.7%–2.9% *n* = 2 [32, 34] *N* = 230	NR	0.01% to 0.4% *n* = 3 [42, 49, 54] *N* = 15,711	NR
Vascular injury	0.6% *n* = 1 [11] *N* = 169	NR	NR	NR	NR	NR	NR
Keloid scar	3.1% *n* = 1 [15] *N* = 479	NR	NR	NR	NR	NR	NR
Persistent skin erythema (>3 days)	NR	NR	NR	NR	NR	1.27% *n* = 1 [49] *N* = 496	NR
Wound infection	1% to 5% *n* = 2 [70] *N* = 519	NR	NR	NR	NR	0% to 0.5% *n* = 3 [42, 49, 54] *N* = 15,711	NR
Malignant hyperthermia	0% to 1.1% *n* = 2 [19, 20] *N* = 967	NR	NR	NR	NR	NR	NR
Persistent pain (>3 days) or excessive intraprocedural pain	NR	NR	1% *n* = 1 [28] *N* = 98	0% *n* = 1 [32] *N* = 128	0.5% *n* = 1 [44] *N* = 444	0.03% to 2.4% *n* = 4 [41, 42, 49, 54] *N* = 15,794	0.7% to 2.3% *n* = 3 [59, 60, 61] *N* = 326
Persistent numbness or hyperesthesia at the biopsy site	NR	NR	1.8% *n* = 1 [27] *N* = 55	NR	NR	0.03% to 0.15% *n* = 2 [42, 54] *N* = 15,215	0.7% *n* = 1 [59] *N* = 149
Persistent weakness	NR	NR	NR	NR	NR	NR	2.3% *n* = 1 [60] *N* = 86
Anxiety or panic episode	NR	5% *n* = 1 [56] *N* = 40	NR	NR	NR	0.8% *n* = 1 [49] *N* = 496	NR
Presyncope or syncope	NR	NR	0.5% *n* = 1 [30] *N* = 220	NR	1.4% *n* = 1 [44] *N* = 444	0.2% *n* = 1 [48] *N* = 379	1.3% *n* = 1 [62] *N* = 160

*Note*: Grey shaded boxes = complication not reported for specific muscle biopsy procedure. Data presented from studies that explicitly reported complications from the procedure.

Abbreviations: *n*, number of studies; *N*, number of biopsies across all indicated studies; NR, not reported.

In general, muscle biopsies were well tolerated by patients, with periprocedure pain being the most frequently reported adverse event. Numerical rating scores of periprocedure pain were between 3.2 and 5.2 (maximum score 10), for various muscle biopsy techniques [25, 26, 35, 63, 67]. Pain persisting for longer than 14 days was infrequently reported [28, 60, 61]. Of the few studies that evaluated the patient experience of the muscle biopsy, a large‐gauge needle biopsy was reported to be more painful than an open surgical biopsy [63], conchotome biopsy [64] and FNB [67].

## DISCUSSION

The results of this systematic literature review demonstrate that muscle biopsy is a safe and generally well‐tolerated investigation that aids the diagnosis of many neuromuscular disorders. Various percutaneous techniques have been reported, and all except the fine‐needle approach yield sample sizes sufficient for histological analysis. Moreover, the diagnostic yield of a moderate‐ to large‐gauge NMB or conchotome biopsy appears equivalent to that of an open surgical biopsy. These findings have important implications for clinical practice in that percutaneous muscle biopsy can be safely performed at the bedside with only local anaesthesia or light sedation. Cardiorespiratory complications of neuromuscular disease are common, and a diagnostic procedure that does not require general anaesthesia reduces the overall risk to the patient. Repeat biopsies to reassess the diagnosis or monitor response to therapy have been demonstrated to be of clinical utility. The rare persistent adverse event following muscle biopsy suggests that serial assessment of muscle histology may be a viable method of monitoring response to therapy either in clinical practice or clinical trials.

The overall diagnostic yield of each muscle biopsy technique was not possible to calculate because of the heterogeneous data available. Heterogeneous study methodologies and patient selection as well as variable outcomes presented precluded any meta‐analysis. Moreover, the medical and surgical specialities involved in requesting, performing and interpreting muscle biopsies vary within and between institutions and this heterogeneity in personnel may influence the diagnostic outcome of muscle biopsy. Variability in muscle processing protocols between laboratories—for instance, the use of a dissection microscope to orient fibres—may additionally affect the quality of the muscle sample. Importantly, more recent diagnostic advances such as genetic and molecular analyses are likely to improve upon the diagnostic yield of muscle biopsies reported in early studies. While the ‘clinical utility’ of biopsies was a frequent endpoint, the definition of a useful test is highly variable depending on the clinical context and question. Measurement of utility based upon a change in diagnosis ignores other important indications for obtaining a tissue sample. These may include confirmation of diagnosis, exclusion of potentially progressive and fatal myopathic conditions, assigning pathogenicity to molecular variants using RNA sequencing, providing functional insights by linking genetic abnormalities to morphological phenotypes and biobanking for research. The few comparative studies included in this review suggest a reduced sensitivity of FNB for the detection of muscle pathology. However, the diagnostic utility of conchotome and moderate‐ to large‐gauge needle techniques appears equivalent to open surgical biopsy.

Few studies included in this review systematically evaluated the role of imaging to select muscle biopsy sites. Certain myopathies, particularly idiopathic inflammatory myopathies, are characterised by patchy muscle involvement, and false negative biopsies may occur as a result of sampling error. Whether imaging may be used to select a representative muscle for histological examination and hence improve the diagnostic utility of muscle biopsies is thus of interest. One study included in this review concluded that a biopsy of an MRI hyperintense muscle had a lower false negative rate than a biopsy of an MRI negative muscle [23]. This concurs with an earlier study of 25 patients (not included in this review because the muscle biopsy technique was not described) that showed the false negative rate of a muscle biopsy is reduced when the biopsy site is selected on the basis of abnormal MRI findings [71]. Aside from MRI, there is growing interest in the role of novel US techniques to identify muscle pathology [72]. Another benefit of sonographically guided biopsy is that critical structures may be visualised in real time, potentially permitting a wider range of muscles to be safely sampled. Larger prospective studies are needed to definitively demonstrate that image‐directed biopsies are superior to biopsies guided by clinical or EMG findings.

This review has limitations. We intentionally used a comprehensive and inclusive search strategy to capture as many articles that reported muscle biopsy techniques as possible. It is possible this review strategy did not identify studies that did not list muscle biopsy in the abstract or keywords. Our review was limited to English‐language studies and full‐length texts, so it is possible that this study did not include pertinent articles published in other languages or data published in conference proceedings. Additionally, the heterogeneous nature of the studies evaluated prevented meta‐analysis of data and pooling of study results. Importantly, the published literature may not reflect real‐world practice or the diagnostic utility of muscle biopsy; therefore, clinical recommendations regarding specific clinical scenarios cannot be made as a result of this systematic literature review alone. To ensure this review was as representative of clinical practice as possible, we elected to include case series and observational studies. Future endeavours may wish to audit institutional practices worldwide to ascertain a truer representation of the scope of muscle sampling techniques. The strength of our conclusions is limited by the heterogeneity of the data available. However, to our knowledge, this is the first systematic review of muscle biopsy techniques and is the largest and most comprehensive comparison of the utility and complications of muscle biopsies to date.

### What does the future hold for muscle biopsies?

Despite the advancements in genetic profiling, autoantibody testing and the increasing sophistication of muscle imaging techniques, muscle biopsy continues to play an important role in the evaluation of myopathies. Additionally, while this review did not specifically evaluate the diagnostic role of genetic and molecular analysis of muscle tissue, it is recognised the advent of high‐throughput, integrative omics technologies has further accelerated our ability to interrogate tissues to an extraordinary level of molecular detail. Although a diagnosis of specific myopathies such as muscular dystrophy or dermatomyositis may no longer require a muscle biopsy [1, 73], it is likely that analysis of affected tissues obtained via muscle biopsy will play an increasingly important role in the era of personalised, omics‐informed neuromuscular medicine. It is foreseeable that molecular signatures obtained from muscle tissue will be increasingly used to understand disease pathogenesis, guide therapeutic decisions and inform individual patient prognostics.

## CONCLUSIONS

Our results demonstrate that the clinical utility of moderate‐ to large‐gauge NMB and conchotome biopsies appears equivalent to that of open surgical biopsies. All muscle biopsy techniques are safe and well tolerated. NMB and conchotome biopsies have the additional benefit of being procedures that can be performed under local anaesthetic at the bedside and do not require a large incision, a surgical team or theatre time. Therefore, given the apparent diagnostic equivalence of all biopsy techniques, NMB or conchotome biopsy could be considered when histopathological evaluation is indicated in cases of suspected myopathy.

## AUTHOR CONTRIBUTIONS

Laura Ross and Jessica Day are responsible for the study conception and design. Laura Ross, Huon Wong and Jessica Day are responsible for the data collection and analysis. Laura Ross, Penny McKelvie, Katrina Reardon, Huon Wong, Ian Wicks and Jessica Day presented the data. Laura Ross and Jessica Day prepared the first draft of the manuscript. Laura Ross, Penny McKelvie, Katrina Reardon, Huon Wong, Ian Wicks and Jessica Day edited the manuscript and approved the final version of the manuscript.

## CONFLICT OF INTEREST STATEMENT

No authors have any conflict of interest to declare.

## ETHICS STATEMENT

Ethical approval was not required for this study.

### PEER REVIEW

The peer review history for this article is available at https://publons.com/publon/10.1111/nan.12888.

## Supporting information


**Data S1.** Index 1: Data extraction template

## Data Availability

Data sharing is not applicable to this article as no new data were created or analysed in this study.
