# Supplementary material for: Muscle biopsy practices in the evaluation of neuromuscular disease: A systematic literature review
Source: Neuropathol Appl Neurobiol. 2023 Feb 20;49(1):e12888. doi: 10.1111/nan.12888 (PMC10946625; doi:10.1111/nan.12888)
Supplement: Supplementary file 1 — Data S1. Index 1: Data extraction template [file NAN-49-0-s001.docx]

**Supplementary Index 1: Data extraction template**

Reference

- First author
- Year
- Country of study

Study characteristics

- Study design
- Patient diagnosis
- Number of patients

Biopsy technique

- Needle muscle biopsy
  - Type of needle
  - Gauge of needle
- Conchotome biopsy
- Open surgical biopsy
- Location of biopsy procedure
- Muscle(s) biopsied
- Selection procedure for muscle biopsy site
  - EMG performed prior to procedure
  - Imaging guided
    - Ultrasound
    - Magnetic resonance imaging

Biopsy results

- Number of biopsy specimens obtained
- Use of anaesthesia
- Size of incision
- Yield of biopsy
  - Size / volume of tissue
  - Number of fibres
  - Weight of tissue sample
  - Definition of abnormal biopsy
  - Number of biopsies with insufficient tissue for sampling
- Histopathological stains performed
- Diagnoses obtained from biopsy results
- Number of contributory biopsies
- Number of non-diagnostic procedures

Post-procedure complications
